# 2D Versus 3D Laparoscopy in Partial Nephrectomy: A Concurrent, Equipment-Allocated Three-Centre Comparison of Precision and Outcomes

**DOI:** 10.3390/curroncol33100614

**Published:** 2026-10-13

**Authors:** Samer Al-Rawashdah, Malik Ayyad

**Affiliations:** Urology Unit, Department of Special Surgery, Faculty of Medicine, Mutah University, Karak 61710, Jordan

**Keywords:** partial nephrectomy, three-dimensional laparoscopy, warm ischaemia time, renorrhaphy, trifecta, renal cell carcinoma, nephron-sparing surgery, depth perception

## Abstract

Background: Warm ischaemia time is the main modifiable determinant of early renal functional loss after partial nephrectomy, and it is governed largely by the speed of intracorporeal suturing. Three-dimensional (3D) laparoscopy restores binocular depth cues and might shorten the reconstructive phase. Earlier comparisons have largely been before-and-after series, in which a secular trend cannot be separated from the optics. Methods: We analysed 209 consecutive laparoscopic partial nephrectomies performed at three private Jordanian hospitals between January 2023 and December 2025, 102 with two-dimensional (2D) and 107 with 3D visualisation. Both systems were in concurrent use throughout, and allocation followed rotation of a shared 3D tower rather than calendar period. Groups were compared with Welch t, Mann–Whitney U, chi-square and Fisher exact tests with Benjamini–Hochberg control of the false discovery rate. Multivariable models with heteroscedasticity-consistent standard errors and stabilised inverse probability of treatment weighting estimated the adjusted effect. Results: Baseline characteristics were balanced (all standardised mean differences below 0.20). The 3D group had shorter warm ischaemia (adjusted beta −4.76 min, 95% CI −6.28 to −3.24), shorter renorrhaphy (−5.06 min), shorter operative time (−12.35 min), lower blood loss and shorter stay. Warm ischaemia of 25 min or less occurred in 90.7% versus 53.9%. Complications, margins and readmission did not differ. Conclusions: With allocation independent of calendar time, 3D visualisation was associated with a faster reconstructive phase and better ischaemia control.

## Simple Summary

Kidney tumours found early can often be removed while the rest of the kidney is left in place. The operation is usually done through small incisions with a camera. A standard camera gives a flat picture, so the surgeon must judge depth from indirect cues such as shadow and instrument size. Newer three-dimensional camera systems give the surgeon back a sense of depth. We compared 209 patients who had part of a kidney removed, 102 operated with a flat view and 107 with a three-dimensional view. Operations done with the three-dimensional system were shorter, the kidney’s blood supply was clamped for less time, blood loss was lower, and patients went home about half a day sooner. Serious complications were uncommon in both groups and did not differ. The clearest gain was in the time spent sewing the kidney closed, the step that decides how long the clamp stays on.

## 1. Introduction

Kidney cancer accounts for roughly 400,000 new diagnoses and close to 175,000 deaths worldwide each year, and its incidence continues to rise while mortality falls slowly [1]. Analysis of the Global Burden of Disease 2021 dataset confirms that renal cell carcinoma was the only one of the three major urological cancers whose age-standardised incidence rose between 2000 and 2021, with an average annual increase of 0.15% [2,3]. Much of that rise reflects incidental detection of small renal masses on cross-sectional imaging performed for other reasons, which has shifted the surgical question away from whether a kidney can be removed and towards how much of it can be kept.

Nephron-sparing surgery is now the reference treatment for cT1 renal masses whenever it is technically feasible, and the European Association of Urology recommends partial nephrectomy over radical nephrectomy in this setting [4,5]. The operation is nonetheless demanding. It compresses tumour excision and reconstruction of the renal defect into a short window during which the renal artery is clamped, and the surgeon must work quickly without sacrificing the margin. Robot-assisted partial nephrectomy has spread rapidly on the strength of wristed instruments and stereoscopic vision, and pooled data indicate acceptable perioperative outcomes even for moderately and highly complex masses [6]. A systematic review restricted to randomised trials found no difference in overall complications between robotic and laparoscopic partial nephrectomy, although the robotic approach showed fewer minor complications and a small advantage in warm ischaemia time [7]. In one multicentre Japanese series, warm ischaemia exceeded 25 min in 52.7% of laparoscopic cases compared with 18% of robotic cases, and trifecta achievement was correspondingly lower after the laparoscopic approach [8]. A single-surgeon twin-centre comparison reached the same conclusion, with shorter warm ischaemia and better early renal function after robotic surgery [9].

Robotic platforms remain unavailable in many health systems for reasons of capital cost, consumable expense and maintenance. For those centres the practical question is not robotic versus laparoscopic, but whether the specific deficit that makes laparoscopic partial nephrectomy difficult can be corrected within a laparoscopic platform. That deficit is stereopsis. Conventional two-dimensional (2D) laparoscopy forces the surgeon to infer depth from motion parallax, relative size and shadow, which is adequate for dissection but costly during suturing, when needle angle and bite depth must be judged repeatedly against a bleeding, moving surface. Three-dimensional (3D) laparoscopy restores binocular disparity while preserving the instrumentation, ergonomics and cost structure of conventional laparoscopy.

Evidence for 3D laparoscopy outside urology is substantial but not uniform. A meta-analysis of six studies of distal gastrectomy reported shorter operative time, less blood loss and shorter hospital stay with 3D systems [10], and a randomised trial with five-year follow-up confirmed that these short-term gains did not come at an oncological cost [11]. A multicentre cohort of 356 adrenalectomies found shorter operative time, lower blood loss, fewer conversions and shorter stay in the 3D group [12]. Against this, a randomised trial of total abdominal colectomy found no difference in operative time despite a clear surgeon preference for the 3D view [13], and among laparoscopic novices the advantage of 3D over 4K 2D disappeared once tasks were crossed over between systems [14]. Work in gynaecology has shown better depth perception and lower surgical strain with 3D, with mixed effects on hard outcomes [15].

Partial nephrectomy is a plausible setting in which the benefit of stereopsis should exceed the average, because the rate-limiting step is intracorporeal suturing under a clock. Direct evidence exists but is thin and methodologically constrained. A meta-analysis of 2D against 3D systems across urology found warm ischaemia significantly shorter with 3D during partial nephrectomy, while operative time, blood loss, dissecting time and suturing time showed no pooled difference [16]. A propensity-matched series of complex tumours reported warm ischaemia of 19 against 27 min and operative time of 105 against 128 min favouring 3D [17]. Both share a design limitation. 3D was adopted after 2D, so calendar period and optics are confounded, and a unit improving for unrelated reasons would produce the same result. Our study differs in that both towers were in concurrent use across the whole period, with allocation set by rotation of a shared 3D tower between three hospitals rather than by date. That removes secular trend and the unit-level learning curve as explanations. Our hypothesis was that 3D visualisation would shorten the reconstructive phase, and thereby the ischaemic interval, without altering margin status or complication rates.

## 2. Materials and Methods

### 2.1. Study Design and Population

This was a retrospective comparative cohort study of consecutive patients who underwent laparoscopic partial nephrectomy for a clinically localised renal mass at three private hospitals in Jordan between January 2023 and December 2025. The three units share a common operative protocol, a single consultant urology team and identical perioperative pathways, which allowed for the records to be pooled [18]. Patients were eligible if they had a unilateral, non-metastatic renal mass amenable to nephron-sparing surgery, complete perioperative records and at least 30 days of follow-up. Planned radical nephrectomy, bilateral or multifocal resection, and open or robot-assisted approaches were excluded. The final cohort comprised 209 patients, 102 operated with a 2D system and 107 with a 3D system.

Allocation was not randomised. It reflected the availability of the 3D tower at each hospital on the day of surgery, since the stereoscopic tower was shared between the three sites on a rotating basis and no case was scheduled around it. Both systems were in continuous concurrent use for the whole study period rather than in sequence, so calendar time and visualisation mode are not confounded. The study is reported in line with the STROBE recommendations for observational research.

All procedures were performed by the same consultant urology team, comprising three consultant urologists. Each had more than 10 years of independent laparoscopic practice and a personal series of more than 100 laparoscopic partial nephrectomies before the study opened. Every surgeon operated with both systems throughout the period. Cases were distributed 35, 34 and 33 between surgeons in the 2D group and 37, 36 and 34 in the 3D group, so no surgeon worked predominantly with either tower.

### 2.2. Surgical Technique and Visualisation Systems

All operations were performed transperitoneally by the same consultant urology team across the three hospitals, using the same instrument set, energy devices and suture materials throughout the study period. The transperitoneal route was used for every case, so that access does not confound the comparison; the retroperitoneal alternative carries its own learning curve and a different operative time profile [19]. The 2D arm used a conventional high-definition laparoscopic tower (Karl Storz Image1 S, Karl Storz SE and Co. KG, Tuttlingen, Germany) with a 10 mm 30-degree telescope. The 3D arm used the stereoscopic version of the same platform (Karl Storz Image1 S D3-Link with a 10 mm dual-channel TIPCAM1 S 3D telescope, Karl Storz SE and Co. KG, Tuttlingen, Germany) and passive polarised eyewear (Karl Storz SE and Co. KG, Tuttlingen, Germany) for the operating team. Both towers drove 32-inch surgical displays (Karl Storz SE and Co. KG, Tuttlingen, Germany) at the same working distance. The optical system was the only element that differed between arms; port placement, hilar dissection, mannitol use and clamping policy were unchanged [20]. Restricting the difference to the display chain matters, because the reported advantage of 3D systems is attributed specifically to the restoration of binocular disparity rather than to any change in instrumentation [14,15].

After mobilisation of the colon and exposure of the hilum, the renal artery was clamped with a laparoscopic bulldog clamp (Aesculap Yasargil, B. Braun, Melsungen, Germany) and the tumour excised with cold scissors (Karl Storz SE and Co. KG, Tuttlingen, Germany) along the pseudocapsular plane. Renorrhaphy was carried out in two layers. The inner layer was a running 3–0 barbed polyglyconate suture (V-Loc 180, Medtronic, Minneapolis, MN, USA) placed across the resection bed, and the outer layer a 0-polyglactin 910 cortical closure (Vicryl, Ethicon, Somerville, NJ, USA) secured with the sliding clip technique using Hem-o-lok polymer clips (Teleflex, Wayne, PA, USA). This combination was chosen because barbed sutures, running closure and sliding clips are the three renorrhaphy elements most consistently associated with shorter warm ischaemia in the published literature [21], and because barbed suture maintains tension between bites without an assistant holding the suture line [22]. The clamp was released after the outer layer. Warm ischaemia time was recorded from clamp application to clamp release, and renorrhaphy time from the first bite of the inner layer to completion of the outer layer, both by the circulating nurse using the theatre clock.

Early unclamping was used throughout. The bulldog clamp was released once the inner layer was complete and haemostasis of the resection bed was satisfactory, and the outer cortical layer was then completed off clamp. Warm ischaemia time was recorded from clamp application to clamp release and therefore ends part way through the reconstruction. Renorrhaphy time was recorded from the first bite of the inner layer to completion of the outer layer and spans both the clamped and the unclamped portion of the closure. The two intervals overlap without one containing the other, which is why renorrhaphy time exceeds warm ischaemia time in 175 of 209 cases, by a mean of 8.2 min. Both were timed by the circulating nurse using the theatre clock.

Preoperative variables were age, sex, body mass index, maximum radiological tumour diameter, R.E.N.A.L. nephrometry score, laterality, dominant polar location, haemoglobin, serum creatinine and estimated glomerular filtration rate. Tumour complexity was scored with the R.E.N.A.L. system on preoperative cross-sectional imaging, which remains the most widely applied of the anatomical classification systems and performs comparably with PADUA, DAP and C-Index for perioperative risk [23]. Estimated glomerular filtration rate was derived from serum creatinine using the 2021 CKD-EPI equation; creatinine-based estimates are imprecise at the level of the individual patient, so the values here are used for group comparison rather than for individual staging [24]. Preoperative estimated glomerular filtration rate is also the dominant predictor of renal function after partial nephrectomy, which is why it was recorded as a baseline covariate rather than as an outcome [25].

Intraoperative variables were operative time, warm ischaemia time, renorrhaphy time, estimated blood loss and any intraoperative adverse event. Postoperative variables were haemoglobin at approximately 48 h, length of index admission, any complication within 30 days, final margin status on permanent section, and unplanned readmission within 30 days. Complications were graded with the Clavien–Dindo system, which is graded by the level of intervention required and has high inter-rater reliability, although it does not capture intraoperative events; those were therefore recorded separately [26].

The co-primary outcomes were warm ischaemia time and renorrhaphy time. Secondary outcomes were operative time, estimated blood loss, haemoglobin drop, hospital stay, intraoperative and 30-day complications, Clavien–Dindo grade III or higher, positive surgical margin and 30-day readmission. Two derived binary outcomes were specified before analysis. The first was warm ischaemia of 25 min or less, a threshold adopted as a trifecta component in several contemporary series [27,28]. The second was trifecta achievement, defined here as the combination of a negative surgical margin, warm ischaemia of 25 min or less, and freedom from any intraoperative or 30-day complication. Tumour complexity was dichotomised at a R.E.N.A.L. score of 8 or higher for subgroup analysis and reported in three conventional strata for descriptive purposes.

### 2.3. Statistical Analysis

Analyses were performed in Python 3.12 (Python Software Foundation; https://www.python.org) using Pandas 3.0 (https://pandas.pydata.org), SciPy 1.17 (https://scipy.org) and statsmodels 0.15 (https://www.statsmodels.org). Figures were drawn with Matplotlib 3.11 (https://matplotlib.org). Distributional assumptions were tested with the Shapiro–Wilk statistic within each group rather than assumed. Continuous variables satisfying normality in both groups were compared with the Welch *t* test, which does not assume equal variances; the remainder were compared with the Mann–Whitney U test. Mean with standard deviation and median with interquartile range are both reported for every continuous outcome, so that readers can judge the effect of that choice. Categorical variables were compared with the chi-square test, or the Fisher exact test where any expected cell count fell below five. Clavien–Dindo grade was additionally analysed as an ordinal variable.

Effect sizes accompany every comparison, because a *p* value alone does not convey clinical relevance. For continuous outcomes we report the mean difference with a Welch–Satterthwaite 95% confidence interval and Hedges g; for binary outcomes, the risk difference and the odds ratio with confidence intervals. Baseline comparability was assessed with standardised mean differences, taking 0.10 as the threshold for meaningful residual imbalance, since significance testing of baseline variables in an observational cohort answers a question about sampling rather than about confounding. Because six continuous and seven categorical outcomes were tested, *p* values within each family were adjusted by the Benjamini–Hochberg procedure and are reported as q values.

Confounding was addressed in two independent ways so that the conclusion would not rest on a single modelling choice. First, multivariable linear regression with HC3 heteroscedasticity-consistent standard errors was fitted for each continuous outcome, and multivariable logistic regression for each binary outcome, adjusting for age, sex, body mass index, tumour size, R.E.N.A.L. score and upper-pole location. Estimated blood loss was additionally modelled on the natural log scale, so that the treatment effect can be read as a ratio of geometric means. Linear model assumptions were checked with the Breusch–Pagan test for heteroscedasticity, the Shapiro–Wilk test on residuals and variance inflation factors. Calibration and discrimination of the logistic model for trifecta were assessed with the Hosmer–Lemeshow statistic and the area under the receiver operating characteristic curve. Second, a propensity score for receiving 3D visualisation was estimated by logistic regression on age, sex, body mass index, tumour size, R.E.N.A.L. score, upper-pole location and laterality, and stabilised inverse probability of treatment weights were derived from it. Balance after weighting was confirmed by weighted standardised mean differences.

Because cases were pooled across three hospitals that shared a protocol and a surgical team, centre was not entered as a random effect; the consequence of that choice is stated in the Limitations. Three secondary analyses were prespecified. Effect modification by tumour complexity and by upper-pole location was tested through multiplicative interaction terms. Sequential adjustment asked whether the reduction in warm ischaemia was explained by faster renorrhaphy, and whether the reduction in hospital stay was explained by lower blood loss, shorter ischaemia and fewer complications; this analysis is descriptive and is not formal causal mediation. Because both systems were used across the same calendar period, operative date was entered as a covariate to check that the group difference was not an artefact of secular improvement in unit performance. Two-sided *p* values below 0.05 were treated as significant, and achieved power was calculated post hoc for the primary outcome.

## 3. Results

### 3.1. Cohort and Baseline Comparability

The cohort comprised 209 patients, 102 in the 2D group and 107 in the 3D group, operated across the three participating hospitals between January 2023 and December 2025. Mean age was 55.5 years, mean body mass index 27.7 kg/m^2^, and 134 patients (64.1%) were men. Mean tumour size was 3.5 (SD 1.0) cm and the median R.E.N.A.L. score was 7 (IQR 6–8). No variable had missing values.

Table 1 shows that the two groups were closely comparable. Every standardised mean difference was below 0.20 and none of the univariate comparisons reached significance. The largest imbalances were in body mass index (27.3 versus 28.1 kg/m^2^, standardised mean difference 0.190, *p* = 0.171) and age (54.5 versus 56.4 years, standardised mean difference 0.185, *p* = 0.183), both favouring a slightly older and heavier 3D group. Tumour size, R.E.N.A.L. score, laterality, polar location, baseline haemoglobin, creatinine and estimated glomerular filtration rate were near-identical. Complexity strata were distributed similarly, with 40, 60 and 2 patients in the low, intermediate and high complexity strata of the 2D group and 37, 64 and 6 in the 3D group (*p* = 0.345). The direction of the residual imbalance matters: an older, heavier group with marginally larger tumours would be expected to fare worse, not better, so any advantage observed in the 3D arm is unlikely to be produced by these differences.

### 3.2. Intraoperative and Early Postoperative Outcomes

**Figure 1:**
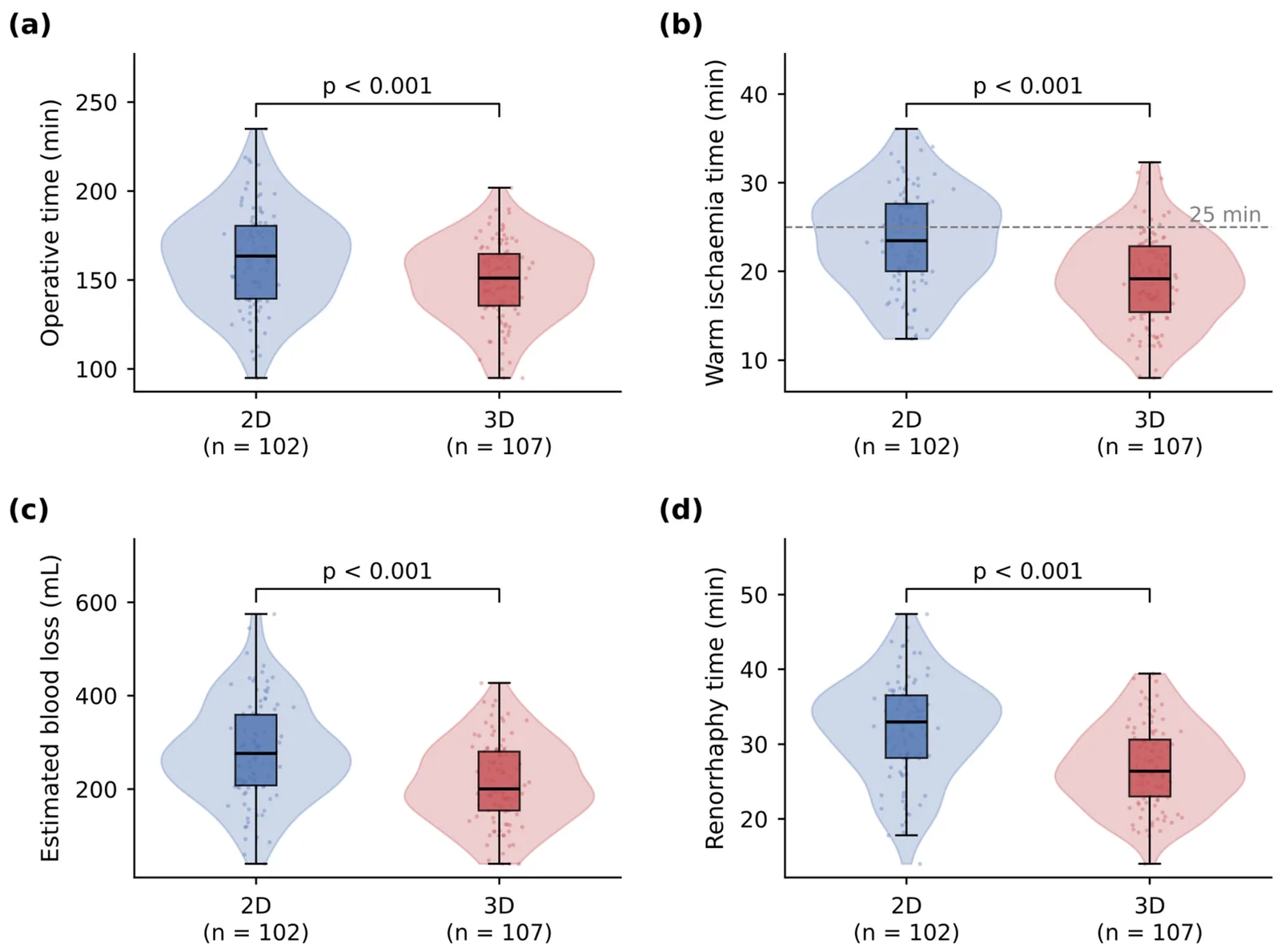
Distribution of key intraoperative outcomes by visualisation group: (**a**) total operative time; (**b**) warm ischaemia time, with the 25 min threshold shown as a dashed line; (**c**) estimated blood loss; (**d**) renorrhaphy time. Violins show the kernel density, boxes the median and interquartile range, whiskers the 1.5 × IQR range, and points the individual cases. *p* values are from the Welch *t* test.

Every continuous perioperative outcome favoured 3D visualisation, and all six remained significant after false discovery rate correction (Table 2, Figure 1). Warm ischaemia time was 23.7 (SD 5.5) min in the 2D group and 19.1 (SD 5.1) min in the 3D group, a mean difference of −4.68 min (95% CI −6.13 to −3.22; q = 3.99 × 10^−9^), with a large effect size (Hedges g −0.88). Renorrhaphy time fell from 32.1 (SD 6.8) to 27.0 (SD 5.5) min, a difference of −5.11 min (95% CI −6.79 to −3.42; g −0.83). The near-equivalence of these two reductions is the central observation of the study: essentially the whole of the ischaemia benefit is accounted for by the reconstructive phase.

Total operative time was 162.2 (SD 27.7) min with 2D and 149.6 (SD 22.4) min with 3D (mean difference −12.61 min, 95% CI −19.50 to −5.73; g −0.50) and estimated blood loss 279.7 (SD 105.6) versus 212.7 (SD 87.2) mL (−67.05 mL, 95% CI −93.55 to −40.56; g −0.69). Haemoglobin drop at 48 h was 1.9 (SD 0.6) versus 1.3 (SD 0.6) g/dL (−0.54 g/dL, 95% CI −0.71 to −0.38; g −0.92). Hospital stay, non-normally distributed in both groups, had a median of 4.2 (IQR 3.5–4.7) days after 2D surgery and 3.6 (IQR 3.0–4.0) days after 3D surgery (q = 1.86 × 10^−6^).

The proportion of patients kept within a warm ischaemia time of 25 min or less rose from 53.9% (55 of 102) to 90.7% (97 of 107), a risk difference of 36.7 percentage points (95% CI 25.6 to 47.9) and an odds ratio of 8.29 (95% CI 3.88 to 17.70; q = 4.51 × 10^−8^). Expressed differently, 47 patients in the 2D group and 10 in the 3D group had ischaemia beyond the 25 min threshold.

### 3.3. Postoperative, Oncological and Safety Outcomes

Categorical safety and oncological outcomes are shown in Table 3. Every complication endpoint moved in favour of 3D, but none reached significance and none survived false discovery rate correction. Intraoperative complications occurred in 12 patients (11.8%) in the 2D group and six (5.6%) in the 3D group (OR 0.45, 95% CI 0.16 to 1.24; q = 0.253), complications within 30 days in 18 (17.6%) and 11 (10.3%) patients (OR 0.53, 95% CI 0.24 to 1.20; q = 0.253), and unplanned readmission in eight (7.8%) and four (3.7%) patients (q = 0.383).

Positive surgical margins were found in six patients (5.9%) after 2D surgery and two (1.9%) after 3D surgery (OR 0.30, 95% CI 0.06 to 1.55; Fisher q = 0.253). Major morbidity was rare in both arms. A single Clavien–Dindo grade IIIa event occurred, in the 3D group, and no patient in either group had a grade IIIb or higher complication. The full grade distribution was 84, 13, 5 and 0 patients across grades 0, I, II and IIIa in the 2D group and 96, 3, 7 and 1 in the 3D group; treated as an ordinal variable, grade did not differ (*p* = 0.177). These comparisons are underpowered and should be read as excluding a large safety penalty rather than as demonstrating equivalence.

**Figure 2:**
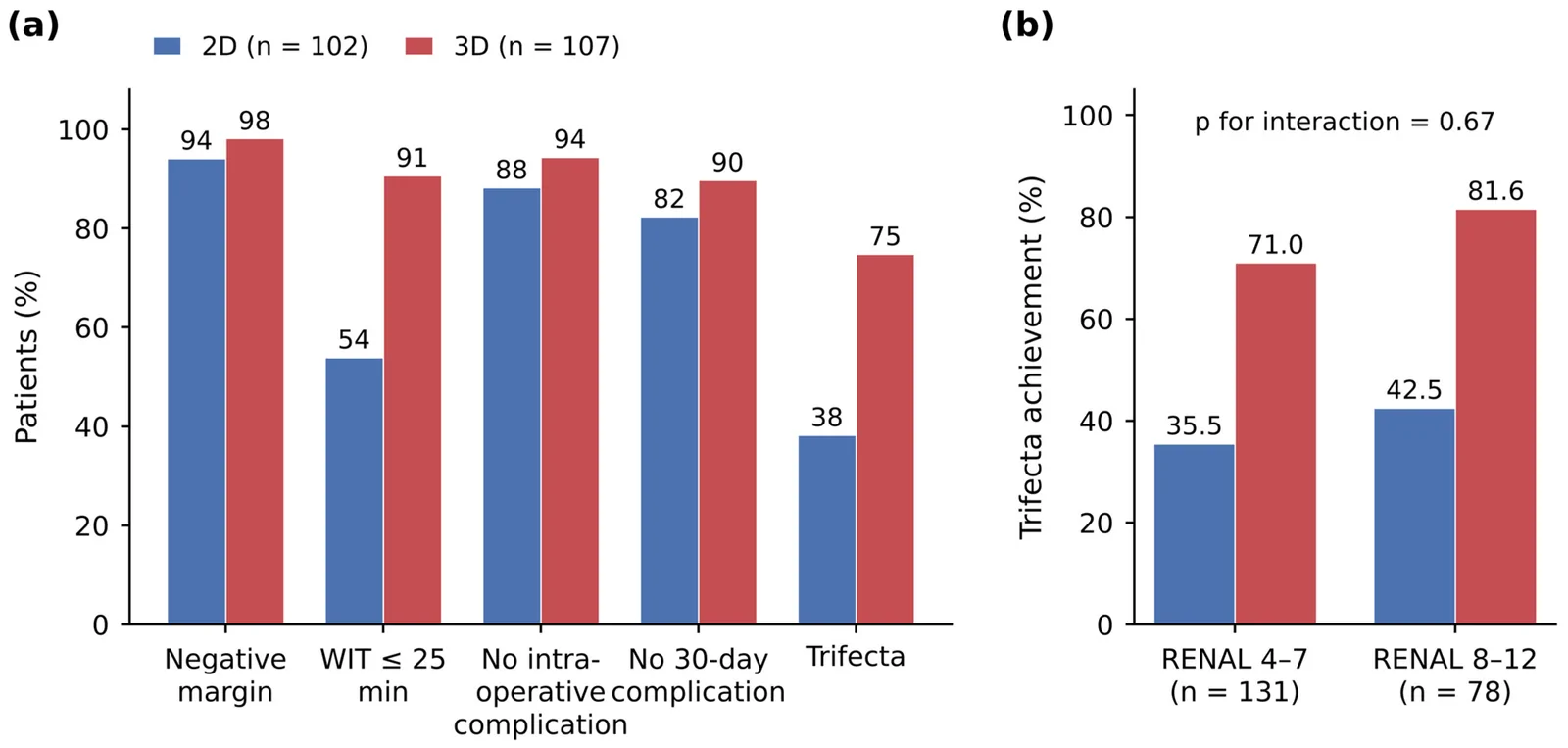
Trifecta achievement by visualisation group: (**a**) the composite endpoint and each of its four components, showing that the between-group gap is driven almost entirely by the warm ischaemia component; (**b**) trifecta achievement stratified by tumour complexity, with the *p* value from a multiplicative interaction term in the adjusted logistic model. WIT: warm ischaemia time.

Trifecta was achieved in 39 of 102 patients (38.2%) after 2D surgery and 80 of 107 (74.8%) after 3D surgery, a risk difference of 36.5 percentage points (95% CI 24.0 to 49.0) and an odds ratio of 4.79 (95% CI 2.65 to 8.65; q = 7.30 × 10^−7^). Figure 2a decomposes the composite and shows that the gap is driven almost entirely by the ischaemia component. The margin component differed by 4 percentage points and the two complication components by 6 and 7 percentage points, whereas the ischaemia component differed by 37 percentage points.

### 3.4. Multivariable and Weighted Analyses

**Figure 3:**
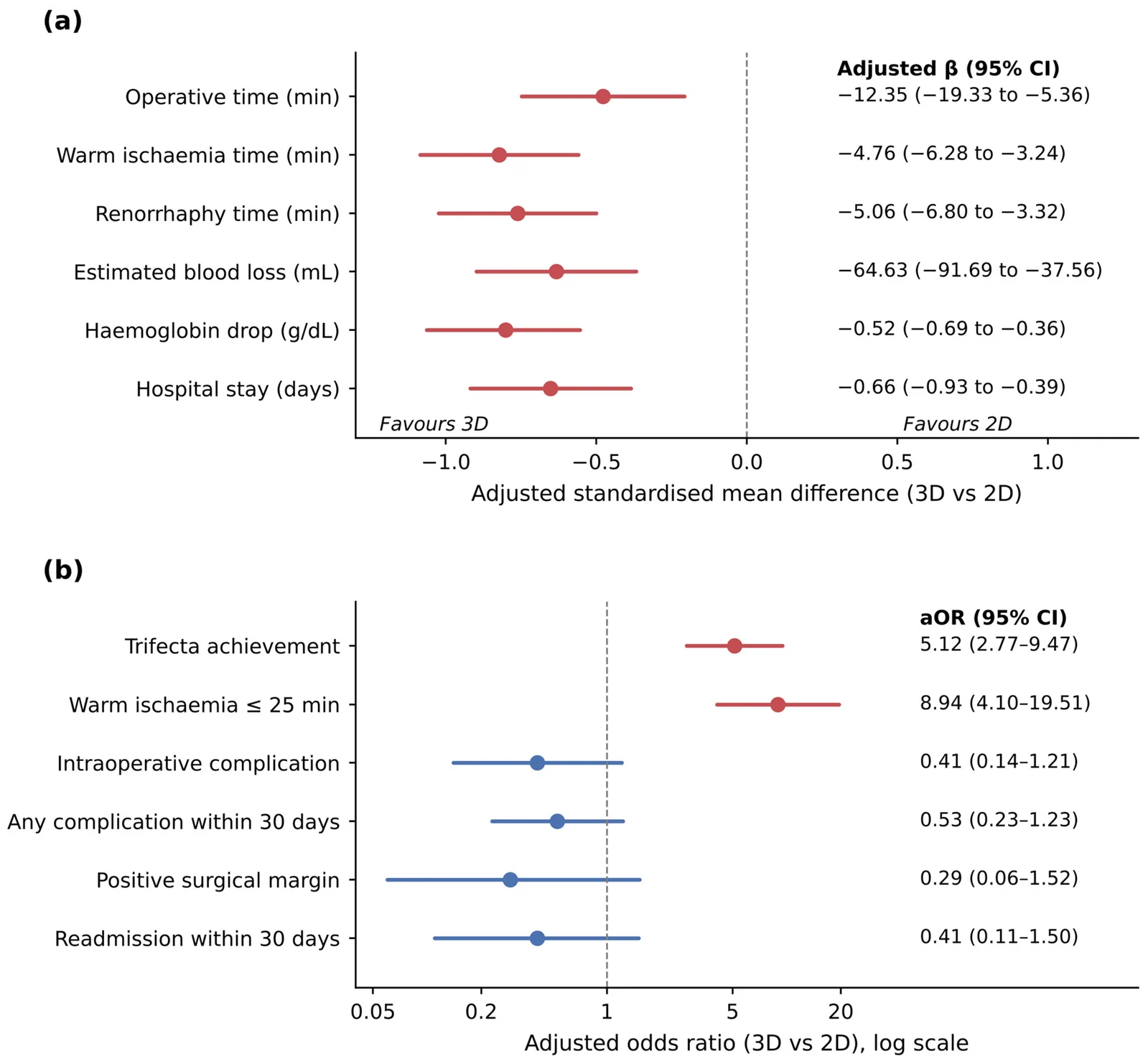
Adjusted effect of 3D versus 2D visualisation across all study outcomes: (**a**) linear models, plotted as adjusted standardised mean differences, with adjusted beta coefficients in outcome units given alongside; (**b**) logistic models, plotted as adjusted odds ratios on a logarithmic scale. All models adjust for age, sex, body mass index, tumour size, R.E.N.A.L. score and upper-pole location. Horizontal bars are 95% confidence intervals.

Adjustment for age, sex, body mass index, tumour size, R.E.N.A.L. score and upper-pole location left every estimate essentially unchanged (Table 4, Figure 3). The adjusted difference in warm ischaemia time was −4.76 min (95% CI −6.28 to −3.24; *p* = 8.2 × 10^−10^), and in renorrhaphy time −5.06 min (95% CI −6.80 to −3.32). Adjusted differences were −12.35 min for operative time, −64.63 mL for blood loss, −0.52 g/dL for haemoglobin drop and −0.66 days for hospital stay. On the log scale, blood loss with 3D was 0.75 times that with 2D (95% CI 0.66 to 0.86; *p* = 4.4 × 10^−5^). Model diagnostics were satisfactory for the primary outcome: the Breusch–Pagan test gave *p* = 0.301, residuals were normally distributed (Shapiro–Wilk *p* = 0.515), and all variance inflation factors were 1.07 or below. Visualisation mode was the only covariate that predicted warm ischaemia time; age, sex, body mass index, tumour size, R.E.N.A.L. score and upper-pole location were all non-significant in that model.

The adjusted odds ratio for trifecta was 5.12 (95% CI 2.77 to 9.47; *p* = 2.0 × 10^−7^), and for warm ischaemia of 25 min or less 8.94 (95% CI 4.10 to 19.51). The trifecta model was adequately calibrated (Hosmer–Lemeshow *p* = 0.14) with modest discrimination (area under the curve 0.729), which is expected when a single dominant predictor is present. Adjusted odds ratios for intraoperative complication, 30-day complication, positive margin and readmission were 0.41, 0.53, 0.29 and 0.41 respectively, all with confidence intervals crossing unity.

The propensity score for 3D allocation had poor discrimination (c-statistic 0.581), which is itself informative: measured characteristics carried almost no information about which system a patient received, consistent with allocation by equipment availability rather than by case selection. Stabilised weights ranged from 0.71 to 1.54 and achieved near-perfect balance, with all weighted standardised mean differences below 0.01. Weighted estimates were almost identical to the regression estimates: −4.76 min for warm ischaemia, −5.04 min for renorrhaphy, −12.54 min for operative time, −64.68 mL for blood loss, −0.53 g/dL for haemoglobin drop, −0.67 days for stay, and an odds ratio of 4.85 (95% CI 2.68 to 8.76) for trifecta. Agreement between two methods resting on different assumptions strengthens the inference that the association is not an artefact of the adjustment strategy.

### 3.5. Subgroup, Sequential and Sensitivity Analyses

The absolute reduction in warm ischaemia time was larger in complex tumours, −6.09 min for a R.E.N.A.L. score of 8 or higher against −3.83 min for scores below 8, but the interaction was not significant (*p* = 0.179), and the study was not powered for it. Trifecta rates rose from 35.5% to 71.0% in the lower complexity stratum and from 42.5% to 81.6% in the higher stratum, with no evidence of effect modification (*p* for interaction = 0.67; Figure 2b). Upper-pole location did not modify the effect on warm ischaemia (*p* for interaction = 0.94).

Sequential adjustment supported the mechanistic reading. Adding renorrhaphy time to the warm ischaemia model attenuated the 3D coefficient from −4.76 to −4.40 min, an attenuation of only 7.7%, indicating that faster suturing and shorter ischaemia are close to the same measurement rather than one causing the other through a separate path. By contrast, the effect on haemoglobin drop was not attenuated at all by adjustment for warm ischaemia and blood loss (−0.52 to −0.54 g/dL), so the haemoglobin difference is not simply a downstream consequence of the measured intraoperative variables. The reduction in hospital stay attenuated by 12.7% after adjustment for complications, blood loss and ischaemia, leaving most of that effect unexplained by the variables recorded here.

Warm ischaemia time did not drift over the study period within either group. Mean values by tertile of operative date were 23.5, 23.1 and 24.5 min in the 2D group and 19.5, 18.4 and 19.2 min in the 3D group. Entering operative date as a continuous covariate left the group coefficient at −4.66 min (*p* = 9.6 × 10^−10^) while the date coefficient was null (*p* = 0.838). The difference between systems therefore does not reflect a learning curve that happened to coincide with adoption of the 3D tower. For the primary outcome the achieved effect size was 0.88 and post hoc power approached 1.00; the cohort had 80% power to detect a standardised difference of 0.39, so genuinely small differences in the rarer categorical outcomes would not have been detected.

## 4. Discussion

In this three-hospital cohort of 209 laparoscopic partial nephrectomies, 3D visualisation was associated with a warm ischaemia time 4.8 min shorter and a renorrhaphy time 5.1 min shorter than 2D, with less blood loss, a smaller haemoglobin drop, a shorter operation and a shorter admission. Complications, Clavien–Dindo grade, margin status and readmission did not differ. Estimates were stable across regression and inverse-probability weighting. Because both towers ran concurrently throughout, the comparison is not open to the secular-trend explanation that applies to before-and-after series.

The most useful finding is not the size of the ischaemia reduction but its location. Renorrhaphy and warm ischaemia fell by almost the same number of minutes, and adding renorrhaphy time to the ischaemia model removed less than a tenth of the treatment effect. Excision, hilar dissection and clamp handling contributed almost nothing [20]. That is what one would predict if the benefit of stereopsis is specific to intracorporeal suturing, where needle angle and bite depth must be judged repeatedly on a curved, bleeding surface without haptic feedback. The pattern matches work elsewhere: 3D gave novices a speed advantage in box-trainer tasks that persisted through the learning curve [14], and in gynaecological cancer surgery it shortened the dissection component specifically while improving rated depth perception [15].

Set against the two published 3D versus 2D comparisons in partial nephrectomy, our estimates are consistent in direction and magnitude. A propensity-matched series of complex tumours reported warm ischaemia of 19 against 27 min, an 8 min advantage, with operative time of 105 against 128 min [17]. Our 19.1 against 23.7 min reproduces almost exactly the 3D figure while showing a smaller absolute gap, which fits a cohort of mostly intermediate rather than high complexity. The pooled urological meta-analysis also found warm ischaemia significantly shorter with 3D during partial nephrectomy but no difference in operative time, blood loss or suturing time [16]. Our operative time and blood loss differences were significant where the pooled estimates were not, which may reflect the larger single-procedure sample or the concurrent design; it may equally reflect that our estimates are unadjusted for surgeon [29]. Taken together, the warm ischaemia finding is now reproduced in three independent cohorts, while the secondary benefits remain unsettled.

Whether a five-minute reduction in warm ischaemia matters clinically depends on where the cohort sits on the ischaemia injury curve, and that question remains unsettled. A meta-analysis of ten studies and 4993 patients concluded that longer warm ischaemia was associated with lower postoperative estimated glomerular filtration rate and poorer short-term renal function, with a threshold for harm between 25 and 30 min [30]. A radionuclide substudy of the CLOCK randomised trial placed the inflection much earlier, at 10 min, but found the effect beyond that point statistically detectable and clinically negligible [31]. A propensity-matched analysis described the relationship as non-linear, rising between 10 and 30 min in patients with normal baseline function and plateauing earlier, between 10 and 20 min, in those with compromised function [32]. A large series comparing warm, cold and no-ischaemia techniques found no difference in relative glomerular filtration rate change at 3 or 12 months [33], and histological analysis of preserved parenchyma years after clamped partial nephrectomy attributed deterioration mainly to hypertension, diabetes and pre-existing chronic kidney disease rather than to the ischaemic insult [34]. Together these findings suggest that our observed reduction is unlikely to translate into a measurable long-term functional gain in patients with normal baseline function, and that its value lies in widening the margin of safety for patients who tolerate ischaemia poorly. Baseline estimated glomerular filtration rate averaged 85 mL/min/1.73 m^2^ in both groups, so this cohort was not enriched for such patients.

The trifecta result should be read narrowly. Trifecta rose from 38.2% to 74.8%, but Figure 2a shows the composite does almost no work beyond restating the ischaemia finding: the margin component differed by 4 percentage points and the two complication components by 6 and 7, against 37 for the ischaemia component. The endpoint here is therefore a redundant reflection of shortened warm ischaemia rather than evidence of broader surgical success, and we do not present it as the latter. This is a known property of the measure. In 186 central tumours prolonged ischaemia was the dominant cause of trifecta failure [28], and in clinical T1b tumours ischaemia beyond 25 min accounted for two thirds of failures against 23.3% for margins and 13.3% for major complications [27]. A composite weighted towards its most frequently violated component moves whenever that component moves. Our 3D rate is nonetheless close to the 74% from two European robotic referral centres [35], although their median ischaemia was 14 min.

The oncological and safety data are reassuring but not conclusive. Positive margins occurred in 5.9% of 2D and 1.9% of 3D cases, within the published range for minimally invasive partial nephrectomy [36,37]. The direction is plausible, since better depth perception should help maintain a consistent plane around the pseudocapsule, and warm ischaemia time has been identified as an independent predictor of positive margins in a multicentre analysis of 2166 robotic partial nephrectomies [38]. With only eight events, however, the margin odds ratio interval spans 0.06 to 1.55 and is compatible with anything from a large benefit to a modest harm. The same applies to complications: every point estimate favoured 3D, none was significant, and the study excludes a large safety penalty from 3D adoption without demonstrating a safety advantage.

Two features of the design bear on how much weight the estimates can carry. The propensity score for 3D allocation discriminated barely better than chance, indicating that measured patient and tumour characteristics did not drive allocation; that is the expected signature of allocation by rotation of a shared tower between the three hospitals, and it is difficult to reconcile with meaningful selection of easier cases into the 3D arm. Second, regression and weighted estimates agree to two decimal places on most outcomes, so the findings are not sensitive to how confounding was modelled. Neither feature addresses unmeasured confounding, and one candidate stands out.

## 5. Limitations

The study is retrospective and non-randomised, so residual confounding cannot be excluded, and four limitations bear directly on the questions a reader will ask of it. First, surgeon-level data were not retrievable case by case from the operative records. All cases were performed by one consultant team, every member of which used both systems throughout, and no surgeon worked predominantly with either tower; but we cannot present a per-surgeon breakdown or adjust for operator. Surgeon experience is the strongest known confounder in partial nephrectomy, and a series of 529 robotic cases showed that surgeon generation alone produced operative time differences of 19 to 24 min at matched volumes [39]. This remains the principal threat to our interpretation. Second, the case-level hospital identifier was not retained in the pooled dataset, so we could not tabulate outcomes by centre or fit a mixed effects model with hospital as a random effect. With a shared protocol, team and instrument set the expected clustering is small, but it is not zero and we cannot demonstrate its absence. Third, renal function was recorded only at baseline. No postoperative creatinine, discharge or 1-month estimated glomerular filtration rate, or acute kidney injury incidence is available, so the clinical value of a 4.8 min ischaemia reduction is inferred from published ischaemia injury data rather than demonstrated here; this is the single most important gap in the study. Fourth, complications were recorded as presence or absence with a Clavien–Dindo grade, without the underlying event type, so we cannot tabulate specific organ injuries, transfusions or urine leaks. The intraoperative rate of 11.8% in the 2D arm cannot therefore be characterised further. Beyond these, follow-up ends at 30 days, so margin status cannot be linked to recurrence; no surgeon-reported workload or eye strain was collected, which matters because 3D carries its own ergonomic cost, with greater eye strain and dizziness reported in a randomised cholecystectomy comparison [40]; our trifecta definition counts all complications rather than only grade III or higher, making it stricter than some published versions; and subgroup analyses were exploratory and underpowered. A prospective study capturing operator, centre and 12-month renal function would answer all four.

The practical implication is narrow but real. For units performing laparoscopic partial nephrectomy without robotic access, and particularly for private and mid-volume centres where the capital and consumable cost of a robotic platform is prohibitive, a 3D tower is a comparatively inexpensive change that addresses the specific perceptual deficit responsible for slow renorrhaphy, and it requires no retraining in a new instrument set [41]. Evidence outside urology suggests the gain is real for suturing-intensive operations and absent for others [11,13], so it should not be generalised to procedures in which reconstruction is not rate-limiting. A randomised trial stratified by surgeon, reporting renal function at 12 months alongside surgeon workload, would settle the question that this cohort can only raise.

## 6. Conclusions

Among 209 laparoscopic partial nephrectomies performed at three Jordanian hospitals, 3D visualisation was associated with a warm ischaemia time 4.8 min shorter and a renorrhaphy time 5.1 min shorter than 2D, with lower blood loss, a smaller haemoglobin drop, a shorter operation and a shorter stay. Warm ischaemia of 25 min or less was achieved in 90.7% of 3D cases against 53.9% of 2D cases. Complications, Clavien–Dindo grade, margin status and readmission did not differ, though the study was not powered for small differences in these. Two features give the estimate more weight than a conventional retrospective comparison. Both visualisation systems were in concurrent use for the whole period, with allocation set by rotation of a shared tower rather than by calendar date, so neither secular trend nor the unit learning curve explains the difference, and the reduction in ischaemia was confined almost entirely to the reconstructive phase, which is consistent with a benefit of stereoscopic vision specific to intracorporeal suturing. The study cannot show that shorter ischaemia improved renal function, because postoperative function was not measured. For units without robotic access, 3D laparoscopy is a plausible route to better ischaemia control, and the hypothesis now requires a prospective trial with surgeon-level stratification and renal function at 12 months.

## Figures and Tables

**Table 1:** Baseline patient and tumour characteristics by visualisation group.

Characteristic	2D Group (*n* = 102)	3D Group (*n* = 107)	SMD	*p*
Age, years, mean (SD)	54.5 (10.0)	56.4 (9.8)	0.185	0.183
Male sex, *n* (%)	66 (64.7)	68 (63.6)	0.024	0.976
Body mass index, kg/m^2^, mean (SD)	27.3 (4.0)	28.1 (3.9)	0.190	0.171
Baseline haemoglobin, g/dL, mean (SD)	13.8 (1.3)	13.7 (1.3)	0.092	0.508
Baseline creatinine, mg/dL, median (IQR)	1.0 (0.9–1.1)	1.0 (0.9–1.1)	0.019	0.964
Baseline eGFR, mL/min/1.73 m^2^, mean (SD)	85.2 (14.2)	85.6 (14.6)	0.024	0.862
Tumour size, cm, mean (SD)	3.4 (1.0)	3.6 (1.0)	0.133	0.338
R.E.N.A.L. score, median (IQR)	7 (6–8)	7 (6–8)	0.027	0.990
Right-sided tumour, *n* (%)	55 (53.9)	58 (54.2)	0.006	1.000
Upper-pole tumour, *n* (%)	36 (35.3)	40 (37.4)	0.043	0.865
Tumour location, *n* (upper/middle/lower)	36/34/32	40/32/35	-	0.867
Complexity, *n* (low/intermediate/high) *	40/60/2	37/64/6	-	0.345

SD: standard deviation; IQR: interquartile range; SMD: standardised mean difference; eGFR: estimated glomerular filtration rate. Continuous variables were compared with the Welch *t* test or the Mann–Whitney U test according to the Shapiro–Wilk result within each group; categorical variables with the chi-square test. * Low: R.E.N.A.L. 4–6; intermediate: 7–9; high: 10–12. An SMD below 0.10 indicates negligible residual imbalance.

**Table 2:** Intraoperative and early postoperative continuous outcomes.

Outcome	2D (*n* = 102) Mean (SD)	3D (*n* = 107) Mean (SD)	2D Median (IQR)	3D Median (IQR)	Mean Difference (95% CI)	Hedges g	*p*	q *
Operative time, min	162.2 (27.7)	149.6 (22.4)	163.4 (139.4–180.4)	151.2 (135.6–164.6)	−12.61 (−19.50 to −5.73)	−0.50	3.9 × 10^−4^	3.9 × 10^−4^
Warm ischaemia time, min	23.7 (5.5)	19.1 (5.1)	23.5 (20.0–27.6)	19.2 (15.4–22.9)	−4.68 (−6.13 to −3.22)	−0.88	1.3 × 10^−9^	4.0 × 10^−9^
Renorrhaphy time, min	32.1 (6.8)	27.0 (5.5)	33.0 (28.2–36.5)	26.4 (23.0–30.6)	−5.11 (−6.79 to −3.42)	−0.83	1.1 × 10^−8^	2.1 × 10^−8^
Estimated blood loss, mL	279.7 (105.6)	212.7 (87.2)	277 (208–359)	201 (155–281)	−67.05 (−93.55 to −40.56)	−0.69	1.3 × 10^−6^	1.9 × 10^−6^
Haemoglobin drop at 48 h, g/dL	1.9 (0.6)	1.3 (0.6)	1.9 (1.4–2.2)	1.2 (1.0–1.7)	−0.54 (−0.71 to −0.38)	−0.92	2.6 × 10^−10^	1.6 × 10^−9^
Hospital stay, days †	4.2 (1.0)	3.6 (0.9)	4.2 (3.5–4.7)	3.6 (3.0–4.0)	−0.68 (−0.94 to −0.42)	−0.71	1.5 × 10^−6^	1.9 × 10^−6^

SD: standard deviation; IQR: interquartile range; CI: confidence interval. Mean differences are 3D minus 2D, so negative values favour 3D. Confidence intervals use the Welch–Satterthwaite approximation. * q values are Benjamini–Hochberg adjusted *p* values across the six outcomes in this family. † Hospital stay was non-normally distributed in both groups (Shapiro–Wilk *p* = 0.024 and *p* = 0.021) and was tested with the Mann–Whitney U test; all other outcomes were tested with the Welch *t* test.

**Table 3:** Categorical perioperative, oncological and safety outcomes.

Outcome	2D (*n* = 102) *n* (%)	3D (*n* = 107) *n* (%)	Risk Difference, Percentage Points (95% CI)	Odds Ratio (95% CI)	*p*	q *
Intraoperative complication	12 (11.8)	6 (5.6)	−6.2 (−13.8 to 1.5)	0.45 (0.16–1.24)	0.181	0.253
Positive surgical margin †	6 (5.9)	2 (1.9)	−4.0 (−9.3 to 1.2)	0.30 (0.06–1.55)	0.163	0.253
Any complication within 30 days	18 (17.6)	11 (10.3)	−7.4 (−16.7 to 2.0)	0.53 (0.24–1.20)	0.180	0.253
Clavien–Dindo grade III or higher †	0 (0.0)	1 (0.9)	0.9 (−0.9 to 2.8)	1.92 (0.06–57.99)	1.000	1.000
Unplanned readmission within 30 days	8 (7.8)	4 (3.7)	−4.1 (−10.4 to 2.2)	0.46 (0.13–1.56)	0.328	0.383
Warm ischaemia time 25 min or less	55 (53.9)	97 (90.7)	36.7 (25.6 to 47.9)	8.29 (3.88–17.70)	6.4 × 10^−9^	4.5 × 10^−8^
Trifecta achievement ‡	39 (38.2)	80 (74.8)	36.5 (24.0 to 49.0)	4.79 (2.65–8.65)	2.1 × 10^−7^	7.3 × 10^−7^

CI: confidence interval. Risk differences are 3D minus 2D. Odds ratios above 1 favour 3D for the last two rows and indicate higher risk with 3D for all other rows. * q values are Benjamini–Hochberg adjusted *p* values across the seven outcomes in this family. † Compared with the Fisher exact test because an expected cell count fell below five; all other rows used the chi-square test. ‡ Negative surgical margin, warm ischaemia time of 25 min or less, and freedom from any intraoperative or 30-day complication. Clavien–Dindo grade distribution (0/I/II/IIIa) was 84/13/5/0 in the 2D group and 96/3/7/1 in the 3D group; analysed as an ordinal variable, *p* = 0.177.

**Table 4:** Unadjusted, multivariable-adjusted and inverse probability-weighted estimates of the effect of 3D visualisation.

Outcome	Unadjusted Effect (95% CI)	Multivariable-Adjusted Effect (95% CI) *	*p*	IPTW-Adjusted Effect (95% CI) †
**Linear models, adjusted beta in outcome units**				
Operative time, min	−12.61 (−19.50 to −5.73)	−12.35 (−19.33 to −5.36)	5.3 × 10^−4^	−12.54 (−19.56 to −5.52)
Warm ischaemia time, min	−4.68 (−6.13 to −3.22)	−4.76 (−6.28 to −3.24)	8.2 × 10^−10^	−4.76 (−6.26 to −3.26)
Renorrhaphy time, min	−5.11 (−6.79 to −3.42)	−5.06 (−6.80 to −3.32)	1.3 × 10^−8^	−5.04 (−6.77 to −3.31)
Estimated blood loss, mL	−67.05 (−93.55 to −40.56)	−64.63 (−91.69 to −37.56)	2.9 × 10^−6^	−64.68 (−91.40 to −37.95)
Estimated blood loss, ratio ‡	-	0.75 (0.66–0.86)	4.4 × 10^−5^	-
Haemoglobin drop, g/dL	−0.54 (−0.71 to −0.38)	−0.52 (−0.69 to −0.36)	2.7 × 10^−10^	−0.53 (−0.69 to −0.36)
Hospital stay, days	−0.68 (−0.94 to −0.42)	−0.66 (−0.93 to −0.39)	2.3 × 10^−6^	−0.67 (−0.93 to −0.40)
**Logistic models, adjusted odds ratio**				
Trifecta achievement	4.79 (2.65–8.65)	5.12 (2.77–9.47)	2.0 × 10^−7^	4.85 (2.68–8.76)
Warm ischaemia 25 min or less	8.29 (3.88–17.70)	8.94 (4.10–19.51)	3.7 × 10^−8^	-
Intraoperative complication	0.45 (0.16–1.24)	0.41 (0.14–1.21)	0.106	-
Any complication within 30 days	0.53 (0.24–1.20)	0.53 (0.23–1.23)	0.139	-
Positive surgical margin	0.30 (0.06–1.55)	0.29 (0.06–1.52)	0.144	-
Readmission within 30 days	0.46 (0.13–1.56)	0.41 (0.11–1.50)	0.177	-

CI: confidence interval; IPTW: inverse probability of treatment weighting. All estimates compare 3D with 2D visualisation. * Adjusted for age, sex, body mass index, tumour size, R.E.N.A.L. score and upper-pole location, with HC3 heteroscedasticity-consistent standard errors for linear models. † Stabilised weights derived from a propensity score modelling age, sex, body mass index, tumour size, R.E.N.A.L. score, upper-pole location and laterality; weights ranged from 0.71 to 1.54 and all weighted standardised mean differences were below 0.01. ‡ Ratio of geometric means from a model fitted on the natural log scale. Model diagnostics for warm ischaemia time: Breusch–Pagan *p* = 0.301, Shapiro–Wilk on residuals *p* = 0.515, all variance inflation factors 1.07 or below, R2 = 0.180. Trifecta logistic model: Hosmer–Lemeshow *p* = 0.14, area under the curve 0.729.

## Data Availability

The data presented in this study are available on request from the corresponding author. The data are not publicly available because they contain information that could compromise the privacy of patients treated at the participating private hospitals.
